# A care team-based classification and population management schema for connected diabetes care

**DOI:** 10.1038/s41746-020-00313-3

**Published:** 2020-08-07

**Authors:** Brian J. Levine, Kelly L. Close, Robert A. Gabbay

**Affiliations:** 1 Close Concerns, San Francisco, CA USA; 2 Joslin Diabetes Medical Center, Harvard Medical School, Boston, MA USA

**Keywords:** Diabetes, Disease prevention

## Abstract

It has been proposed that telehealth may help to combat the epidemic of diabetes and other chronic diseases in the US. As a result of rapid technological advancement over the past decade, there has been an explosion in virtual diabetes management program offerings rooted in smartphone technology, connected devices for blood glucose monitoring, and remote coaching or support. Such offerings take many forms with unique features. We provide a care team-based classification system for connected diabetes care programs and highlight their strengths and limitations. We also include a framework for how the different classes of connected diabetes care may be deployed in a health system to promote improved population health.

Despite significant advances in the diabetes therapeutic toolkit in the past 15 years, diabetes prevalence, cost to the economy, and population-level outcomes are not improving. There are an estimated 34.2 million people with diabetes in the US (10.5% of the population)^1^, the disease cost the country $327 billion in 2017^2^, and a recent study suggested that the percentage of people with diabetes who have achieved cardiovascular risk factor control did not improve between 2005 and 2016^3^. [Note that risk factor control may have improved since 2016, as many diabetes drugs have since shown to be cardioprotective in cardiovascular outcomes trials.] Still, poor access to high-quality diabetes care may be one of the drivers behind these concerning statistics^4–6^.

In many ways, the diabetes field has been an early adopter and innovator in the chronic disease digital medicine space. This is due to the high demand (high disease prevalence), high measurability of the primary disease biometric (blood glucose), and high potential for behavior change given the short feedback loop between behavior and outcome (change in blood glucose).

There is an opportunity for connected diabetes care—virtual diabetes management programs rooted in smartphone technology, connected devices for blood glucose monitoring, and remote coaching or support—to fill gaps in care access and quality^7^. Accordingly, there has been an explosion in connected diabetes care offerings in recent years. As it stands, however, there is a paucity of extensive high-quality clinical evidence supporting these programs. There are few robust randomized controlled trials, though a number are in progress^7^. To date, connected care programs have more commonly reported group differences in HbA1c in pre-post analyses in the absence of comparator groups^7–9^. One reason for this observation may be the extended timeline of prospective, randomized outcomes trials, which does not align well with the iterative digital health development cycle. Pragmatic, observational, and registry studies with real-world data will be key to fully assessing the benefits of specific connected care offerings. Still, as these programs evolve, they have the potential to drive better population diabetes outcomes by: (1) filling temporal and comorbid gaps in care; (2) decentralizing and scaling expert care; (3) providing self-management support to reinforce healthy behaviors; and (4) using a data-driven approach to recommend therapies (i.e., medications, technologies, and programs) to the right person at the right time. Of equal importance, as the COVID-19 pandemic has illuminated, people are increasingly seeking remote care^10^ and systems must be implemented that are effective, efficient, and satisfying.

The explosion of connected diabetes care has produced an array of products with various features, strengths, limitations, and target users. Particularly in light of the COVID-19 outbreak and rapid, likely irreversible shift to virtual care delivery^10^, it is important that providers, health plans, employers, and people with diabetes arrive at a shared understanding and vocabulary for discussing connected diabetes care. This paper provides a classification and population management schema in an effort to increase awareness around the current landscape of connected diabetes care programs. We have sourced examples and inputs for this classification largely from the commercial connected diabetes care landscape because startup organizations have developed a plethora of qualitatively varying models and because they are built for scale and longevity. Scholars have argued that it may be advantageous for health systems to be “early adopter customers” rather than builders of “promising clinically disruptive businesses,”^11^ but there are lessons to be learned from the landscape for any potential builder or buyer.

## Classification schema for connected diabetes care

A handful of different connected diabetes care models have emerged. We have divided them into the following five categories according to their healthcare provider (HCP) personnel resources and therefore scalability and care options: (1) Virtual diabetes clinics with a medical doctor (MD), physician assistant (PA), and/or nurse practitioner (NP), (2) non-physician clinician-driven (NPCD) connected care, (3) artificial intelligence (AI) health coach, (4) software tools for brick-and-mortar clinicians, and (5) quantified self solution (QSS). The purpose of this classification is purely explanatory and not hierarchical; but each category is likely suited for a different type of patient depending on her present clinical needs and preferences (Table 1). Following the discussion of each model, we provide a non-exhaustive list of examples from today’s landscape.

**Table 1:** Classification of connected diabetes care programs.

	Care team composition	Estimated relative cost	Likely target user	Examples
Virtual diabetes clinic	All clinicians, including physicians, nurse practitioners, and physician assistants	Highest	Highest-risk, highest-cost patients who are furthest from target and need frequent therapeutic adjustments	Virta, Onduo
NPCD	All clinicians, excluding physicians, nurse practitioners, and physician assistants	High	High-risk patients who (a) have access to high-quality, frequent in-person care and/or (b) are on a fairly stable and successful therapeutic regimen	Livongo Health, mySugr, Omada Health, One Drop, Cecelia Health (soon aims to be Virtual Diabetes Clinic), Vida Health, Noom, Dariohealth, Canary Health
AI coach	Automated coaching	Low	Members of large employer/health plans who prefer daily engagement/coaching and do not need frequent therapeutic adjustments	Welldoc, Lark Health
Software tools	Brick-and-mortar clinicians	Low	Everyone with a diabetes or prediabetes diagnosis and a brick-and-mortar clinician	Glooko, Tidepool, Fitbit (Twine Health), device manufacturer software, specific EHR modules
QSS	None	Middle	Motivated people who may be at risk of developing disease	UHC Motion Program, Devoted Health Medicare Advantage, Aetna Attain

### Virtual diabetes clinics

The archetypal virtual diabetes clinic aims to approximate the offerings of a brick-and- mortar clinic through connected devices and smartphone-based logging/data capture and interactions. Virtual diabetes clinics provide medical services and have prescribing clinicians—MDs, PAs, NPs, and, in some cases, pharmacists—on the care team that can adjust medications. In addition, they may have non-prescribing providers such as certified diabetes care and education specialists (CDCES’s) and psychosocial experts. As a result of this staffing and licensing, virtual diabetes clinics may be able to optimize medication therapy, reduce therapeutic inertia, and prescribe prescription-only medical devices such as continuous glucose monitoring (CGM) systems, while other connected diabetes care providers rely on the patient’s primary care provider (PCP) or endocrinologist to make such therapeutic changes. Virtual diabetes clinics can also deploy non-prescription connected devices and tools such as blood glucose meters (BGMs), weight scales, and blood pressure cuffs, as well as smartphone apps for meal, activity, medication, sleep, and mental health tracking, to enable remote monitoring. A virtual diabetes clinic can allow clinicians to manage a more geographically diverse (and possibly larger) patient panel, with the intended result of improved therapeutic regimens for patients and eased burdens and improved wellbeing for PCPs.

Physical examinations of feet and eyes are still necessary in-person today, though there are already a number of commercialized technologies to monitor for foot ulcers (e.g., Podimetrics^12^), and researchers aspire to develop reliable retinopathy assessments from non-mydriatic smartphone camera images in the not-too-distant future^13^. Even with these developments, most people with diabetes will benefit from occasional in-person visits in addition to participation in a virtual clinic.

A readily apparent downside to the virtual diabetes clinic is its high cost compared to the models described below. Labor for MDs, NPs, and PAs is not inexpensive, nor is the infrastructure and operations required to maintain pan-state licensure, malpractice insurance, and taxation status for a provider organization. The Interstate Medical Licensure Compact, currently an agreement between 29 states, the District of Columbia, and the Territory of Guam, provides a means of licensing individual physicians to practice across many but not all state lines^14^. It does not, however, cover all 50 states nor relieve the maintenance burden on an organization as a whole. In light of the COVID-19 pandemic, the Centers for Medicare and Medicaid Services (CMS) and some states have lifted a number of restrictions on telemedicine^15^. Some, if not most, of these changes may remain in place after the pandemic has subsided, easing the burden of delivering and maintaining a telemedicine organization.

The additional costs of a virtual diabetes clinic compared to entirely bricks and mortar care may be offset by reduced travel-related spending and time (as demonstrated in a Veterans Affairs program^16^) and potentially larger provider panel sizes enabled by technology (this will need to be demonstrated). Ultimately, savings may come through reduced emergency, inpatient, and outpatient utilization, although there is limited evidence to date that this is the case. Given the financial and operational demands of a virtual diabetes clinic today, it is likely best suited to treat the highest-risk and/or -cost patients who are further from treatment targets and require frequent changes in treatment in between their clinic visits.

Examples: Virta Health, Onduo

### NPCD connected diabetes care

NPCD connected care is the most common, and the most commercially successful, variety of connected diabetes care to date^7^. These offerings are similar to virtual clinics, with the exception that they lack medical licensure and prescribing providers (MDs, NPs, PAs, and pharmacists) on the care team. They therefore cannot directly optimize medication therapy or prescribe prescription-only medical devices, but focus on self-management support, lifestyle interventions, and adherence.

Care programs typically consist of a smartphone app, live clinician coaches (typically CDCES’s, registered dietitian nutritionists, or trained health coaches), and non-prescription connected devices. Connected BGMs are the most commonly provided device, though wireless scales, blood pressure cuffs, and activity trackers are increasing being deployed. CGM data can be seamlessly integrated in many programs if patients have an existing prescription, but NPCDs do not prescribe or distribute CGMs. Some NPCD offerings do not include a smartphone app or connected devices and are primarily focused on providing patients with remote phone or internet access to clinicians or peers (moderated by a clinician).

There seems to be commercial interest in expanding NPCD programs. For example, mySugr was acquired by Roche in 2017 for ~$85 million and Livongo Health raised more than $350 million in an initial public offering in 2019^17,18^.

The NPCD model is less costly to maintain than a virtual diabetes clinic and is designed to provide a similar degree of continuous human support. NPCDs may be best suited for people with diabetes who are high risk and high cost but (a) have access to high-quality, frequent in-person care and/or (b) are on a fairly stable and successful therapeutic regimen. It is recommended that all NPCD program member see a brick- and-mortar physician for prescriptions and other therapeutic needs, and that the physician is abreast of the patient’s interactions with the NPCD provider.

Some NPCD connected diabetes care organizations have opted to partner with established, large-scale telemedicine providers such as Teladoc, Doctor on Demand, and MDLive^19–21^. This arrangement may offer the possibility of non-physician clinicians quarterbacking the bulk of care and referring out for a telemedicine consult when necessary, allowing for medication optimization and medical device prescription similar to that seen in a vertically integrated virtual diabetes clinic. This presumably decreases cost of operations for the connected care organization but may reduce the level of care coordination, quality, and continuity as the consulting physician is not fully ingrained in the patient’s care team. It is unclear if there will be a difference in the quality of care provided or patient outcomes between these two strategies, though we look forward to future analyses comparing relevant quality metrics between the two approaches for similar populations.

Examples: Livongo Health, mySugr, Omada Health, One Drop, Cecelia Health (soon aims to be Virtual Diabetes Clinic), Vida Health, Noom, Dariohealth, Canary Health

### AI coach

AI coaches are apps that leverage algorithms and machine learning to provide real-time feedback to users based on data procured through connected devices and manual entry. If there is a human care team, these coaches seldom interact with users. In many cases, the app will provide automated reminders for users to perform self-management tasks such as taking medications, exercising, drinking water, or checking their glucose. Following data acquisition, the app will provide education, reminders, personalized feedback, or feedback designed to be motivational, such as “Your blood glucose is just a little high. Try going for a 20-minute walk.” The more advanced AI coach apps tout the ability to learn users’ behavioral patterns and tailor content and types of interactions to produce the greatest engagement and outcomes. In many of these apps, users may interact directly with a chatbot to ask questions about their personal data or self-management. Automated coaching apps that include insulin dosing modules require a prescription.

The ideal AI coach is highly scalable, responsive, and inexpensive to operate. For this reason, they may be well-suited for large member groups of employers or health plans, and they may work best among more homogenous patient populations. At an individual level, they may be recommended for users who have access to excellent and frequent in-person care but prefer daily engagement/coaching or to interact primarily with a chatbot over a human care team. For this model to be successful, users of AI coaches should still regularly see a physician for prescriptions and other therapeutic needs, and the physician should be abreast of the users’ interactions with the AI coach.

Examples: Welldoc, Lark Health

### Software tools for brick-and-mortar HCPs

Software tools for brick-and-mortar HCPs provide clinicians who practice independently or in a clinic or hospital setting a means of remotely managing, risk-stratifying, and/or communicating with patients. Data included in the tool may be derived from a mobile app, connected device, physician notes, and/or lab values. The most useful features found in these tools are population risk stratification modules, two-way secure communication with patients and other members of the care team, and a consolidated hub for all disease-specific patient data for a clinician to inspect during and between clinic visits. Risk stratification dashboards highlight the patients that are in most need of outreach or a therapeutic adjustment based on metrics such as high A1c, low time-in-range, frequent hyper- or hypoglycemia, lack of self-monitored glucose data, or high diabetes distress. As laid out by Crossen et al.^22^, clinicians can then reach out to subsets of patients by phone, text, video and/or invite for an in-person visit based on their individualized needs.

Developers of these tools are beginning to bolster their products with more prescriptive decision support that recommends specific therapy adjustments rather than simply flagging patients for assessment and outreach. Such software could enable non-endocrinologists to manage diabetes therapies and devices with greater confidence and facility.

Software tools can help brick-and-mortar clinicians more effectively manage populations of people with diabetes. They also provide a secure, low-hassle means of monitoring and communicating with patients between clinic visits. Software tools can also potentially help HCPs to expand their patient panel, though probably not to a significant enough extent that dedicated remote care teams are no longer needed. There are, however, three notable challenges with these software tools: (1) Health systems need to develop efficient workflows that ensure all clinicians practice at the top of their licenses and that physician and NP time is optimally leveraged to serve a large panel size; (2) Not all are currently embedded in the electronic health record (EHR), forcing clinicians to navigate multiple interfaces; and (3) Time spent using these tools is not currently reimbursed but can generate additional work for the provider team (in their current form). Payment reform toward a more capitated or outcomes-based system could allow health systems to reorganize staff, care delivery, and compensation to incentivize optimal utilization of these software tools.

Examples: Glooko, Tidepool, Fitbit (Twine Health), device manufacturer software, diabetes-specific EHR modules

### The quantified self solution (QSS)

QSS’s are the least clinical and most patient-facing of the connected diabetes care solutions. In most QSS’s, health plans or employers provide people with non- prescription connected devices—activity trackers, wireless blood pressure cuffs, wireless scales, etc.—but no coaching. They may include engagement tactics such as gamification, financial incentives, or other motivation.

QSS’s can be excellent tools for some individuals—likely those who are currently more self-motivated and engaged in their self-care—and are likely less expensive than a coaching-intensive intervention. A payer or health system could monitor the data and recommend that certain at-risk participants who are not engaged or helped by the QSS join a more intensive disease prevention or management program. However, evidence that QSS’s have sufficient adherence^23^ or improve outcomes is lacking at this point, save perhaps for home blood pressure monitoring^24,25^.

Examples: UnitedHealthcare Motion program, Devoted Health Medicare Advantage, Aetna Attain

## Toward a patient-centered connected care neighborhood for population management

**Fig. 1:**
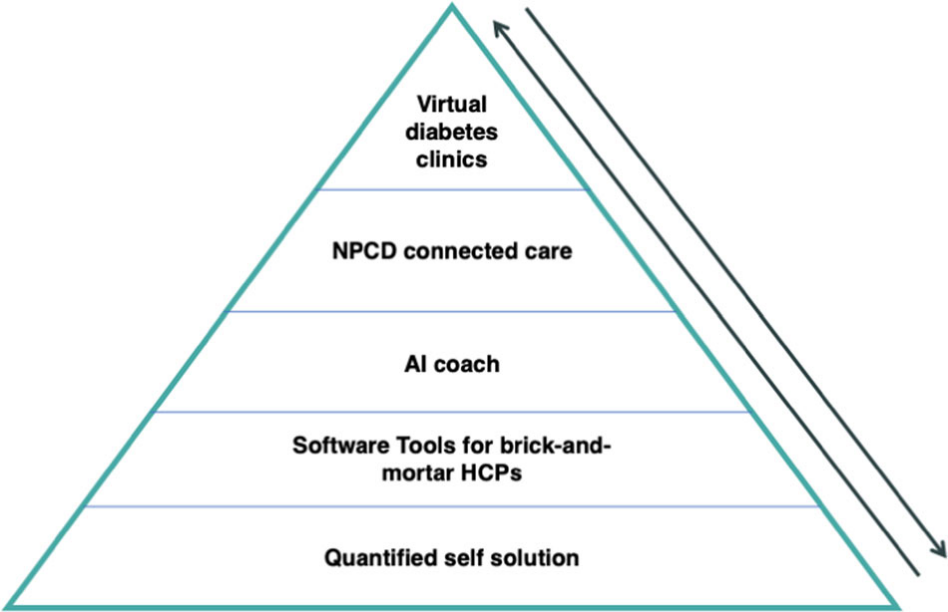
Theoretical risk stratification pyramid showing how the different categories of connected diabetes care could be deployed to improve population health. People can be guided to escalate and de-escalate among the different categories based on clinical need and individual preference.

A health system could leverage the five categories of connected diabetes care to ensure that every person with or at risk of diabetes is receiving the level of care that he or she wants and needs. Figure 1 depicts one way they could be used in concert: Everyone who wants to can receive connected devices to try to stay healthy. Those already at-risk of or diagnosed with diabetes can be added to the software tool of his/her brick-and-mortar clinicians. Individuals who would benefit from some coaching can be provided an AI coach; if they express a desire to interact with a human or would benefit from expert human care and education, then they are escalated to NPCD connected care. Finally, if they require frequent interactions with an MD/NP/PA/pharmacist, medication adjustments, or prescription devices, then they can be escalated to a virtual diabetes clinic.

Members are moved along the rungs of the pyramid as a function of preference and clinical need. Note that these different levels of connected care could be provided by a single vertically integrated organization or platform or by many different ones. The important piece is ensuring that people are guided to escalate and deescalate along the care continuum to ensure all aspects of the triple aim—care quality, patient satisfaction, and cost to the patient and health system—are met. Care navigation and escalation protocols are nothing new and have worked very successfully in some health care settings, but applying them to a largely virtual environment for management of a chronic condition would open the door to more scalable, successful care at a population level.

It is worth noting that certain populations who are at higher risk for type 2 diabetes—the elderly and lower socioeconomic status groups, in particular—may have lower access to or less comfort using technology commonly employed in connected diabetes care programs. These technologies include cellphones (especially smartphones) and internet/broadband. For example, only 53% of people ages 65+ own a smartphone vs. 92% of those ages 30–49; similarly, 71% of people who earn less than $30,000 annually own a smartphone vs. 95% of those earning $75,000+^26^. An organization implementing a patient-centered connected care neighborhood for population diabetes management should consider these populations and offer in-person care, device training, or even devices themselves in order to facilitate equitable treatment quality.

In order to fully realize the benefit of connected diabetes care technology and programs, an organization must establish an integrated care team that includes the patient’s PCP and integrate clinical information into existing workflows in the EHR. These actions unfortunately represent major barriers to implementation as they run counter to current incentives, both explicit (e.g., fee-for-service, “facility fees”, EHR fragmentation) and implicit (e.g., cultural, social, and political biases toward the status quo)^27^. However, increasing adoption of bundled and capitated payments should encourage a shift to technology-enabled, population-based care delivery models such as that proposed in Fig. 1.

## Conclusion

In the face of COVID-19, clinics have embraced telemedicine as a legitimate form of health care encounter^28^. Meanwhile, over the past decade-plus, the world of connected diabetes care has demonstrated that adoption of remote care enables far more than the simple replacement of in-person MD visits with video visits. Rather, the unique types of connected diabetes care variably employ multidisciplinary care teams, automation, and device and software connectivity with the intention of better supporting people with diabetes when they are not in the doctor’s office. We are hopeful that this new classification and population management schema for connected diabetes care will help push toward: (1) greater awareness among providers, health plans, employers, and people with diabetes of these models, their strengths and weaknesses; (2) more rigorous research; and (3) widespread implementation of successful models.
